# Systematic camera trapping survey for terrestrial vertebrates in Xuan Lien Nature Reserve, Vietnam

**DOI:** 10.3897/BDJ.12.e135746

**Published:** 2024-10-17

**Authors:** Thanh Van Nguyen, Anh The Luu, Hung Viet Pham, Ha Manh Nguyen, Tam Anh Pham, Mai Thi Nguyen, Minh Duc Le, Anh Tuan Nguyen

**Affiliations:** 1 Central Institute for Natural Resources and Environmental Studies, Vietnam National University, Hanoi, Hanoi, Vietnam Central Institute for Natural Resources and Environmental Studies, Vietnam National University, Hanoi Hanoi Vietnam; 2 Center for Nature Conservation and Development, Hanoi, Vietnam Center for Nature Conservation and Development Hanoi Vietnam; 3 Xuan Lien Nature Reserve, Thanh Hoa, Vietnam Xuan Lien Nature Reserve Thanh Hoa Vietnam; 4 Hong Duc University, Thanh Hoa, Vietnam Hong Duc University Thanh Hoa Vietnam; 5 Faculty of Environmental Sciences, University of Science, Vietnam National University, Hanoi, Hanoi, Vietnam Faculty of Environmental Sciences, University of Science, Vietnam National University, Hanoi Hanoi Vietnam

**Keywords:** hunting threats, monitoring, muntjac, small carnivores

## Abstract

**Background:**

Xuan Lien Nature Reserve was established in 1999 to protect important habitats and wildlife in the northern part of the Annamites in Vietnam. While Xuan Lien is home to many threatened species, it has experienced a high level of human disturbance over the last decades. To document and provide baseline data on the status and distribution of the terrestrial vertebrate fauna community in the region, we conducted a systematic camera trapping survey in Xuan Lien Nature Reserve in 2023. The data collected during the survey will help design proper conservation measures to better conserve the remaining species.

**New information:**

Our study investigates and updates the species richness of mid- to large-sized terrestrial vertebrates, thus providing essential information for developing conservation strategies in Xuan Lien Nature Reserve, Vietnam. As camera traps were set up in a grid-based design, our survey also generated the first-ever systematic data for terrestrial vertebrate fauna in the area. The study covers approximately 21,000 hectares (about 77% of the area), using 35 camera trap stations; each station consists of two cameras. In total, the final dataset consists of 6,276 trap nights, recording at least 46 species in 39 genera. The results suggest that Xuan Lien is a key stronghold for small carnivores, based on the diversity of such species groups in the Reserve. We failed to detect the previously documented Roosevelt’s muntjac (*Muntiacusrooseveltorum* Osgood, 1932) and we only documented a single record of the northern red muntjac (*Muntiacusvaginalis* Boddaert, 1785). Our survey confirms the severity of overhunting and other anthropogenic threats to the mammal fauna, especially ungulates, in the Reserve.

## Introduction

Camera trapping has been widely used recently to survey the terrestrial vertebrate fauna in Vietnam due to its efficiency in the detection of terrestrial animals (Nguyen et al. 2019, Nguyen et al. 2020, Tilker et al. 2020a). Particularly in the last decade, systematic grid-based design for camera-trapping studies has become a method of choice amongst researchers in the region. The grid-based system allows scientists to statistically estimate the probability of occupancy and other important parameters of studied communities rather than report the presence or absence of interesting species (Abrams et al. 2018, Tilker et al. 2020a, Tilker et al. 2020b). Since its first use in Vietnam in the early 2010s, many protected areas in the central and southern regions of Vietnam have employed systematic camera traps to provide baseline data and monitor important species populations (Tilker et al. 2020b). However, its usage in protected areas from Thanh Hoa Province to the north of the country has been much less common (Willcox et al. 2014, Nguyen et al. 2022). Hence, information on the population and distribution of vulnerable vertebrate species in northern Vietnam has been severely limited.

Xuan Lien Nature Reserve, Thanh Hoa Province, Vietnam, is located in the northern part of the Annamites with approximately 27,000 hectares of forested area. Together with the nearby Pu Hoat Nature Reserve in Nghe An Province, it forms a contiguous protected landscape of around 115,000 hectares (Trai et al. 1999). The most recent field survey on mammal diversity in Xuan Lien was conducted in 2011 - 2012 and the main methods were transect survey, night survey and small mammal traps (Phuong et al. 2013). The survey provided a list of 80 mammal species in Xuan Lien. A subsequent genetic analysis survey using hunter's and trophy samples added one more mammal species, the small-toothed palm civet (*Arctogalidiatrivirgata* Gray, 1832) (Giang et al. 2016). Our study is the first to employ a systematic camera survey to assess the terrestrial vertebrate community in Xuan Lien Nature Reserve. We aim to provide baseline data that can be used for future conservation initiatives in Xuan Lien and surrounding regions.

## General description

### Purpose

The survey is conducted to document the occurrence and distribution of terrestrial vertebrates in Xuan Lien Nature Reserve, Thanh Hoa, Vietnam, using a camera trap survey. The survey follows a repeatable, systematic grid-based design with the intention of providing baseline data and establishing a Camelot (Hendry and Mann 2018) monitoring database to support future conservation initiatives and evaluate the effectiveness of protection activities in the area.

## Project description

### Title

Systematic camera trapping survey for terrestrial vertebrates in Xuan Lien Nature Reserve, Vietnam.

### Study area description

Xuan Lien Nature Reserve is located in Thanh Hoa Province, Vietnam and it is located in the northern Annamite landscape. The Reserve was formally established in 1999 and there have been a number of biodiversity surveys in Xuan Lien since the late 1990s. It reportedly still supports several rare and threatened mammals, such as the gaur (*Bosgaurus* C.H. Smith, 1827), the northern pygmy slow loris (*Xanthonycticebusintermedius* Dao Van Tien, 1960), the Asiatic black bear (*Ursusthibetanus* G. Cuvier, 1823), the Indochinese grey langur (*Trachypithecuscrepusculus* Elliot, 1909), the northern white-cheeked gibbon (*Nomascusleucogeny* Ogilby, 1840), the Owston’s civet (*Chrotogaleowstoni* Thomas, 1912) and the stump-tailed macaque (*Macacaarctoides* I.Geoffroy Saint-Hilaire, 1831) (Trai et al. 1999, Phuong et al. 2013, Giang et al. 2016). However, populations of the species in Xuan Lien have substantially declined due to illegal logging and hunting (Trai et al. 1999, Hai et al. 2016). Surprisingly, in 2014, genetic and camera-trapping surveys revealed that the Roosevelt’s muntjac (*Muntiacusrooseveltorum* Osgood, 1932), an enigmatic and extremely elusive species that had only been recorded in Laos, also occurred in the Reserve (Le et al. 2014).

### Funding

This research has been done under the research project QG.22.72 of Vietnam National University, Hanoi.

## Sampling methods

### Sampling description

**Figure 1.**
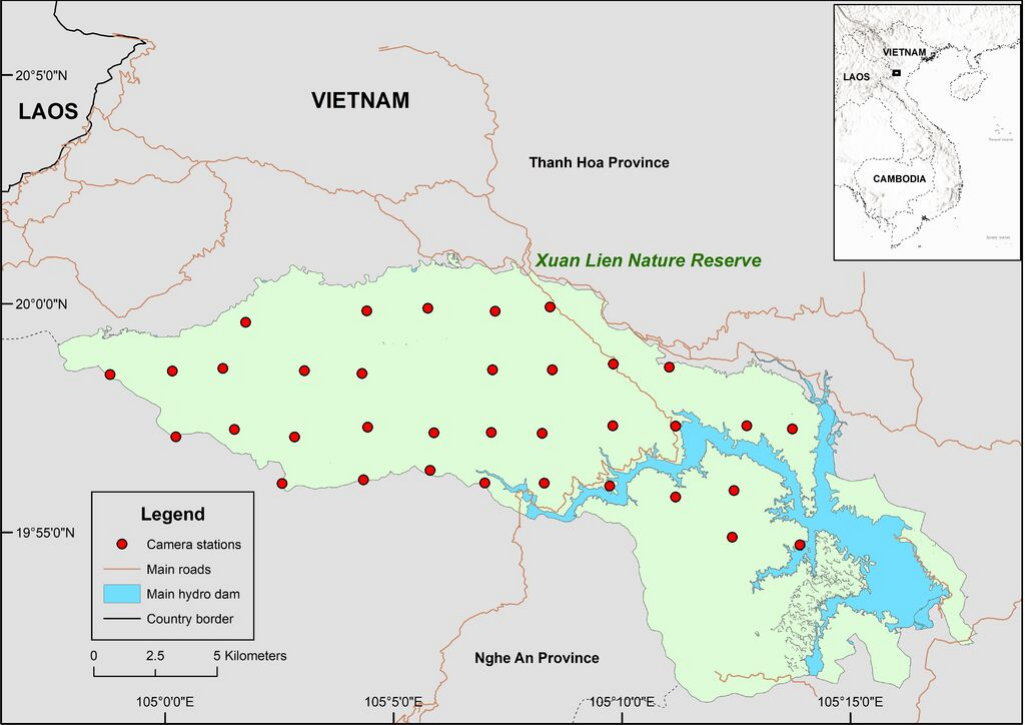
Camera station locations in Xuan Lien Nature Reserve.

Our survey was conducted from February 2023 to June 2023. The survey followed a systematic grid-based camera trapping design developed by Abrams et al. (2018). We intended to survey the entire Reserve area; however, due to the operation of the Cua Dat hydropower plant in the south-eastern part of the Reserve and the subsequent raised water level of the Chu River since 2010, some areas of Xuan Lien have been isolated for around 15 years. Subsequently, our survey only covered about 77% of the Reserve. However, the core zone of the Reserve was fully covered by our survey and such areas were believed to still harbour a high density of vertebrates (Phuong et al. 2013, Hai et al. 2016) (Fig. 1).

In total, we deployed 70 camera traps at 35 locations, which were spaced about 2.5 km apart with a maximum acceptable deviation of 10% (approximately 250 m). Hence, the minimum distance between any camera station was 2.0 km. However, at one station, both cameras were lost and, therefore, we were only able to retrieve 68 cameras at 34 stations. Each camera-trap station consisted of two non-facing camera-trap devices (Reconyx Hyperfire) that were set within a 20 × 20 m plot. Cameras were placed on tree trunks 20–40 cm above the ground and were set up to take three bursts of photos per trigger without delay, recording the date and time of each photo. All cameras were set at a high sensitivity level and were working continuously during the survey. The cameras were deployed for an average of 92.2 ± 2.3 days.

### Quality control

To maximise wildlife records, we configured all cameras to be activated by motion and the sensitivity was set at very high level. Each camera took three photos per trigger, with picture interval in rapid-fire mode, which means the cameras took multiple shots in rapid succession. All cameras were set to run continuously with no rest period and the flash output was at high level.

To ensure the correct scientific name and common name of species, the taxonomic nomenclature followed the Catalogue of Life and GBIF Backbone Taxonomy. In addition, we used the IUCN Red List of Threatened Species and Vietnam Red Data Book 2007 to check the species' conservation status (Ministry of Science and Technology of Vietnam and Vietnamese Academy of Science and Technology 2007). The identification of these species was originally performed and confirmed by two experts who are experienced with identifying Annamite terrestrial animal camera-trap images. Next, all identified images were double-checked and re-confirmed by other independent species experts. Afterwards, all ambiguous records that could not be identified at the genus level were excluded from the final analysis. The records belonging to Muridae were thus removed from the dataset as we were unable to identify them at the species level. Detected domestic fowl, domestic dogs, domestic cattle and human records were also excluded.

### Step description

Collected data were copied into folders named by camera and station codes. Each photo was then identified and moved to a corresponding species sub-folder. The completely identified camera dataset was then checked and analysed using the camtrapR package (Niedballa et al. 2016) in R (R Core Team 2024) to generate a basic statistical report for the collected data. Detections from the two camera devices at the same station and within a 60-minute threshold are considered a single independent detection. Naïve occupancy was calculated as a proportion of the number of camera stations occupied by one particular species and the total number of camera stations (Rovero et al. 2014).

## Geographic coverage

### Description

Xuan Lien Nature Reserve, Thanh Hoa Province, Vietnam.

### Coordinates

19.854 and 20.021 Latitude; 104.956 and 105.301 Longitude.

## Taxonomic coverage

### Description

Mammals, birds and reptiles were identified to the species level, whenever possible.

### Taxa included

**Table:** 

Rank	Scientific Name	Common Name
class	Mammalia	Mammals
class	Aves	Birds
class	Reptilia	Reptiles

## Temporal coverage

### Notes

2023-2-27 - 2023-6-09

## Usage licence

### Usage licence

Other

### IP rights notes

Creative Commons Attribution Non-Commercial (CC-BY-NC 4.0).

## Data resources

### Data package title

Systematic camera trapping survey for terrestrial vertebrates in Xuan Lien Nature Reserve, Thanh Hoa Province, Vietnam.

### Resource link

https://doi.org/10.15468/ns65r2

### Alternative identifiers

https://www.gbif.org/dataset/62cff18a-116e-4582-834d-9e9b3f08722d

### Number of data sets

1

### Data set 1.

#### Data set name

Systematic camera trapping survey for terrestrial vertebrates in Xuan Lien Nature Reserve, Thanh Hoa Province, Vietnam.

#### Data format

Darwin Core Archive

#### Download URL

https://cloud.gbif.org/asia/archive.do?r=2023xuanlienct_01

#### Data format version

Version 1

#### Description

The dataset is published on the Global Biodiversity Information Facility platform, GBIF (Nguyen and Le 2024). It includes all observations of a species where classification was possible. Observations of humans, domestic fowl, domestic dogs and domestic cattle are excluded from the dataset. It is structured as an occurrence dataset, formatted according to the recommendations of the Darwin Core Archive. In this dataset, each row corresponds to an independent detection.

**Data set 1.** 

Column label	Column description
occurrenceID	Unique identifier of the record.
basisOfRecord	The specific nature of the data record.
occurrenceStatus	A statement about the presence or absence of a taxon at a location.
eventDate	Date and time when the occurrence occurred.
kingdom	The full scientific name of the kingdom in which the taxon is classified.
scientificName	The full scientific name, with authorship and date information, if known.
taxonRank	The taxonomic rank of the most specific name in the scientificName.
decimalLatitude	The geographic longitude at which the occurrence took place.
decimalLongitude	The geographic longitude at which the occurrence took place.
geodeticDatum	Spatial reference system upon which the geographic coordinates given in decimalLatitude and decimalLongitude are based.
countryCode	ISO code of the country in which camera location occurs.
dataGeneralizations	Generalisation measures taken to make the shared coordinates less specific than in their original form. High quality data are available upon reasonable request.
class	Full scientific name of the class in which the taxon is classified.
family	Full scientific name of the family in which the taxon is classified.
genus	Full scientific name of the genus in which the taxon is classified.
coordinateUncertaintyInMeters	The horizontal distance (in metres) from the coordinates given in decimalLatitude and decimalLongitude describing the smallest circle containing the whole of the location.
individualCount	The number of individuals present at the time of the occurrence.
organismQuantity	A number or enumeration value for the quantity of the organism.
organismQuantityType	The type of quantification system used for the quantity of organismQuantity.
dynamicProperties	IUCN Red List status of the taxon.
minimumElevationInMeters	The lower limit of the range of elevation in metres.
maximumElevationInMeters	The upper limit of the range of elevation in metres.

## Additional information

### Results

**Figure 2.**
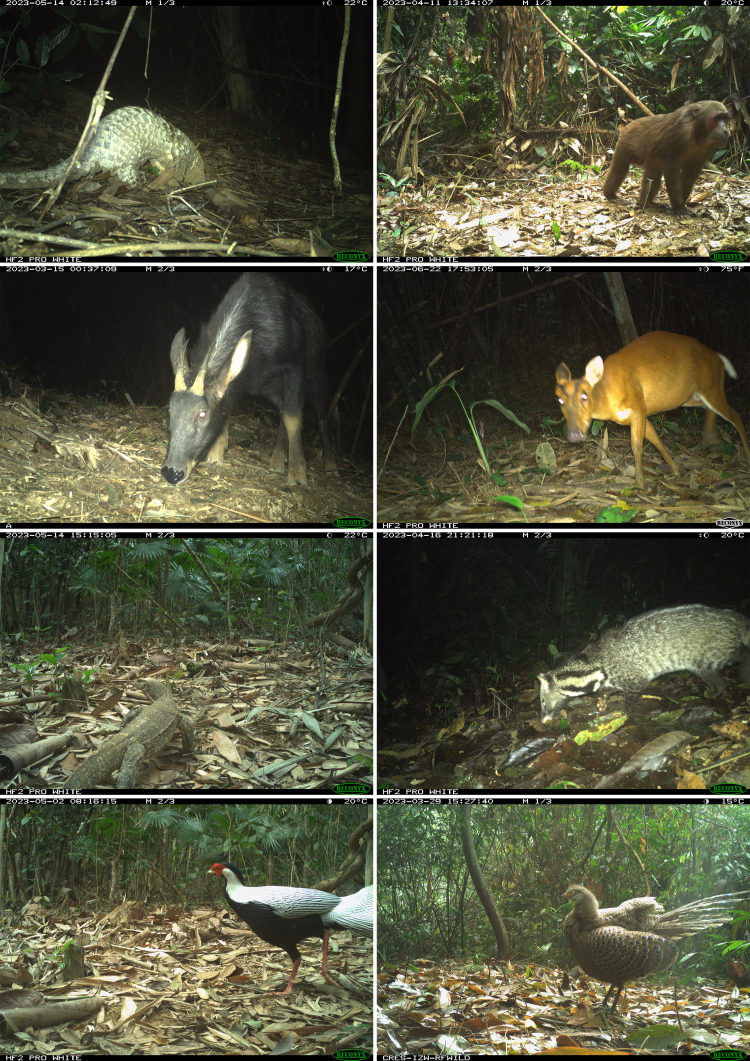
Examples of records from our survey at Xuan Lien Nature Reserve, Thanh Hoa Province, Vietnam. From top left, clockwise: the Sunda pangolin (*Manisjavanica*), the stump-tailed macaque (*Macacaarctoides*), the northern red muntjac (*Muntiacusvaginalis*), the large Indian civet (*Viverrazibetha* Linnaeus, 1758), the grey peacock-pheasant (*Polyplectronbicalcaratum* Linnaeus, 1758), the silver pheasant (*Lophuranycthemera* Linnaeus, 1758), the Asian water monitor (*Varanussalvator* Laurenti, 1768) and the Chinese serow (*Capricornismilneedwardsii*).

A total of 1,002 independent records of at least 46 species, including 21 mammals, 24 birds and one reptile, were documented in our survey. For mammals, 19 taxa were identified to species level and two taxa were only identifiable to genus level (Table 1). Amongst the detected families, Phasianidae had the highest species richness (six species). The survey revealed the presence of three macaque species of the family Cercopithecidae, including the stump-tailed macaque (*Macacaarctoides*), the Assamese macaque (*Macacaassamensis* McClelland, 1840) and the rhesus macaque (*Macacamulatta* Zimmermann, 1780). The species with the highest occurrence records were the ferret badger (*Melogale* I.Geoffroy Saint-Hilaire, 1831 spp.), the yellow-throated marten (*Martesflavigula* Boddaert, 1785) and the Asian palm civet (*Paradoxurushermaphroditus* Pallas, 1777), with naïve occupancy of 0.765, 0.471 and 0.5, respectively (Table 1). Additionally, we recorded several rare species with high conservation value, including the Critically Endangered Sunda pangolin (*Manisjavanica* Desmarest, 1822) (Challender et al. 2019), the Vulnerable mainland serow (*Capricornismilneedwardsii* David, 1869) and the Vulnerable stump-tailed macaque (*Macacaarctoides*) (Fig. 2).

Interestingly, we documented a number of various small carnivore species, including the large Indian civet (*Viverrazibetha* Linnaeus, 1758), the masked palm civet (*Pagumalarvata* C.E.H. Smith, 1827), the spotted linsang (*Prionodonpardicolor* Hodgson, 1841), the Asian palm civet (*Paradoxurushermaphroditus*), the crab-eating mongoose (*Herpestesurva* Hodgson, 1836), the leopard cat (*Prionailurusbengalensis* Kerr, 1792) and the yellow-throated marten (*Martesflavigula*) (Table 1). Their relative commonness in Xuan Lien Nature Reserve highlights the Reserve's high diversity of small carnivores, especially civets. In contrast, we only obtained a single record of the northern red muntjac (*Muntiacusvaginalis* Boddaert, 1785) and we did not record the Roosevelt’s muntjac (*Muntiacusrooseveltorum*) during the survey. Furthermore, four species that had IUCN conservation status of near threatened and above recorded in our survey included the stump-tailed macaque (*Macacaarctoides*), the Assam macaque (*Macacaassamensis*), the Chinese serow (*Capricornismilneedwardsii*) and the Sunda pangolin (*Manisjavanica*). All records of those species came from 14 camera stations in mostly undisturbed habitats of primary evergreen forests, with little to no sign of human activities nearby. The smallest distance from any of those sites to the nearest main roads or human settlements was at least 2.5 km. Four stations that documented the majority of the records (60.4%) for those species were all near or on the ridgeline of the mountain range. Hence, it is likely that remoteness and terrain difficulty play an important role in protecting the remaining threatened taxa in Xuan Lien.

### Discussion

The most recent field survey on mammal diversity for Xuan Lien Nature Reserve was published in 2013 (Phuong et al. 2013) using a combination of transect survey, night survey and traps. The study provided a list of 80 mammal species in Xuan Lien. A subsequent study using molecular approaches confirmed an additional mammal species, the small-toothed palm civet (*Arctogalidiatrivirgata*) (Giang et al. 2016). Hence, the number of documented mammal species from our survey accounts for approximately 31% of the previously reported mammal species in Xuan Lien. Our survey did not document any new species, but validated previous mammal records via camera trap image data for the first time. Additionally, camera trap stations that record the greatest number of mammals were deployed in the south-western part of the Reserve, which is also the most remote region in Xuan Lien (Fig. 1).

Amongst our records, the Sunda pangolin is the least expected species, as Xuan Lien falls within the distribution range of the Chinese pangolin (*Manispentadactyla* Linnaeus, 1758) (Phuong et al. 2013, Challender et al. 2019a, Challender et al. 2019b). Two pangolin experts rigorously verified the Sunda pangolin in our study. Nevertheless, its detection in our study might be a result of unwarranted releasing activities, as suggested by the Reserve staff. Furthermore, two muntjac species, including the northern red muntjac and the Roosevelt’s muntjac, are known to occur in Xuan Lien (Phuong et al. 2013, Le et al. 2014). As our camera stations were set in the elevation range of 150–1,300 m, within the known elevation range of the species (Tilker et al. 2020b, Nguyen et al. 2021) and our survey covered 77% of the Reserve, the lack of Roosevelt’s muntjac record suggests that, if the species still persists, it may occur at a very low density. Additionally, the fact that we only documented a single record of the northern red muntjac, which is naturally more resilient to hunting pressure and is more widely distributed across various forest habitats in Vietnam (Nguyen et al. 2021, Alexiou et al. 2022), raises serious concerns over potential overhunting and habitat loss threats in the Reserve.

During our survey, we documented several signs of ongoing human disturbances inside the Reserve, including four illegal logger camps, small mammal traps and snare lines. Some of those activities were spotted deep inside the Reserve and interviews with local people revealed that exploitation of forest products, while illegal, still happened occasionally in Xuan Lien. Accessibility to the Reserve from the dam and the road systems may facilitate illicit activities (Fig. 1). Therefore, regular patrols and law enforcement activities will play an important role in protecting the remaining species in Xuan Lien Nature Reserve.

## Figures and Tables

**Table 1.** Recorded species during the survey in Xuan Lien Nature Reserve in 2023.

Class	Family	Species	IUCN	Vietnam Red Data Book 2007	Independent detection	Recorded station	Naïve occupancy
Mammalia	Tupaiidae	*Tupaiabelangeri* Wagner, 1841	LC		29	14	0.412
	Cercopithecidae	*Macacaarctoides* I.Geoffroy Saint-Hilaire, 1831	VU	VU	42	11	0.324
		*Macacaassamensis* McClelland, 1840	NT	VU	4	2	0.059
		*Macacamulatta* Zimmermann, 1780	LC	LR	4	2	0.059
	Mustelidae	*Martesflavigula* Boddaert, 1785	LC		20	16	0.471
		*Melogale* spp. I.Geoffroy Saint-Hilaire, 1831	LC		257	26	0.765
		*Lutra* sp. Brisson, 1762			1	1	0.029
	Viverridae	*Pagumalarvata* C.E.H. Smith, 1827	LC		6	3	0.088
		*Paradoxurushermaphroditus* Pallas, 1777	LC		31	17	0.500
		*Viverrazibetha* Linnaeus, 1758	LC		21	9	0.265
	Herpestidae	*Herpestesurva* Hodgson, 1836	LC		36	14	0.412
	Felidae	*Prionailurusbengalensis* Kerr, 1792	LC		22	13	0.382
	Prionodontidae	*Prionodonpardicolor* Hodgson, 1841	LC	VU	16	7	0.206
	Suidae	*Susscrofa* Linnaeus, 1758	LC		15	11	0.324
	Cervidae	*Muntiacusvaginalis* Boddaert, 1785	LC	VU	1	1	0.029
	Bovidae	*Capricornismilneedwardsii* David, 1869	NT	EN	3	3	0.088
	Hystricidae	*Hystrixbrachyura* Linnaeus, 1758	LC		10	7	0.206
		*Atherurusmacrourus* Linnaeus, 1758	LC		1	1	0.029
	Sciuridae	*Callosciuruserythraeus* Pallas, 1779	LC		15	6	0.176
		*Dremomysrufigenis* Blanford, 1878	LC		20	5	0.147
	Manidae	*Manisjavanica* Desmarest, 1822	CR	EN	4	2	0.059
Aves	Phasianidae	*Arborophilachloropus* Blyth, 1859	LC		19	2	0.059
		*Arborophilabrunneopectus* Blyth, 1855	LC		13	6	0.176
		*Arborophilarufogularis* Blyth, 1849	LC		9	5	0.147
		*Gallusgallus* Linnaeus, 1758	LC		49	15	0.441
		*Lophuranycthemera* Linnaeus, 1758	LC	LR	49	12	0.353
		*Polyplectronbicalcaratum* Linnaeus, 1758	LC		27	8	0.235
	Columbidae	*Chalcophapsindica* Linnaeus, 1758	LC		38	7	0.206
		*Pittasoror* R.G.W.Ramsay, 1881	LC		40	19	0.559
		*Pittaelliotii* Oustalet, 1874	LC		29	5	0.147
	Corvidae	*Dendrocittaformosae* Swinhoe, 1863	LC		2	1	0.029
		*Cissahypoleuca* Salvadori & Giglioli, 1885	LC		1	1	0.029
	Dicruridae	*Dicrurusmacrocercus* Vieillot, 1817	LC		2	1	0.029
	Turdidae	*Zootheracitrina* Latham, 1790	LC		8	5	0.147
		*Zootheradauma* Latham, 1790	LC		32	10	0.294
	Muscicapidae	*Myophonuscaeruleus* Scopoli, 1786	LC		101	6	0.176
		*Larvivoracyane* Pallas, 1776	LC		1	1	0.029
		*Copsychusmalabaricus* Scopoli, 1786	LC		1	1	0.029
	Leiotrichidae	*Garrulaxcastanotis* Ogilvie-Grant, 1899	LC		3	2	0.059
		*Garrulaxleucolophus* Hardwicke, 1816	LC		2	2	0.059
	Pellorneidae	*Pellorneumtickelli* Blyth, 1859	LC		10	5	0.147
		*Schoeniparusrufogularis* Mandelli, 1873	LC		1	1	0.029
	Timaliidae	*Erythrogenyshypoleucos* Blyth, 1844	LC		1	1	0.029
	Scolopacidae	*Scolopaxrusticola* Linnaeus, 1758	LC		1	1	0.029
	Ardeidae	*Gorsachiusmelanolophus* Raffles, 1822	LC		1	1	0.029
Reptilia	Varanidae	*Varanussalvator* Laurenti, 1768	LC	EN	4	4	0.118
